# Can Na^18^F PET/CT bone scans help when deciding if early intervention is needed in patients being treated with a TSF attached to the tibia: insights from 41 patients

**DOI:** 10.1007/s00590-020-02776-2

**Published:** 2020-09-05

**Authors:** Henrik Lundblad, Charlotte Karlsson-Thur, Gerald Q. Maguire, Marilyn E. Noz, Michael P. Zeleznik, Lars Weidenhielm

**Affiliations:** 1 Department of Molecular Medicine and Surgery, Karolinska Institute, 171 76 Stockholm, Sweden; 2 School of Electrical Engineering and Computer Science, KTH Royal Institute of Technology, Stockholm, Sweden; 3 Department of Radiology, New York University, New York, NY USA; 4 School of Computing, College of Engineering, University of Utah, Salt Lake City, UT USA

**Keywords:** NaF-18 bone scans, PET/CT, Taylor Spatial Frame, Complex tibia fractures, Tibia osteotomies, Orthopedic surgery

## Abstract

**Purpose:**

To demonstrate the usefulness of positron emission tomography (PET)/computed tomography (CT) bone scans for gaining insight into healing bone status earlier than CT or X-ray alone.

**Methods:**

Forty-one prospective patients being treated with a Taylor Spatial Frame were recruited. We registered data obtained from successive static CT scans for each patient, to align the broken bone. Radionuclide uptake was calculated over a spherical volume of interest (VOI). For all voxels in the VOI, histograms and cumulative distribution functions of the CT and PET data were used to assess the type and progress of new bone growth and radionuclide uptake. The radionuclide uptake difference per day between the PET/CT scans was displayed in a scatter plot. Superimposing CT and PET slice data and observing the spatiotemporal uptake of ^18^F^−^ in the region of healing bone by a time-sequenced movie allowed qualitative evaluation.

**Results:**

Numerical evaluation, particularly the shape and distribution of Hounsfield Units and radionuclide uptake in the graphs, combined with visual evaluation and the movies enabled the identification of six patients needing intervention as well as those not requiring intervention. Every revised patient proceeded to a successful treatment conclusion.

**Conclusion:**

Numerical and visual evaluation based on *all the voxels in the VOI* may aid the orthopedic surgeon to assess a patient’s progression to recovery. By identifying slow or insufficient progress at an early stage and observing the uptake of ^18^F^−^ in specific regions of bone, it might be possible to shorten the recovery time and avoid unnecessary late complications.

**Electronic supplementary material:**

The online version of this article (10.1007/s00590-020-02776-2) contains supplementary material, which is available to authorized users.

## Introduction

The Ilizarov-derived circular Taylor Spatial Frame™ (TSF; Smith & Nephew, Memphis, TN, USA) [1, 2] has the ability to correct deformity in six dimensions and thus has added possibilities to treat difficult fractures and osteotomies [3]. Computed tomography (CT), planar X-ray imaging, and clinical examinations are presently used to evaluate bone healing. However, these techniques are currently unable to predict the healing potential either preoperatively or during treatment. In contrast, a positron emission tomography (PET)/CT Na^18^F bone scan might be a useful addition. Since the ^18^F^−^ ion in blood is absorbed onto the bone surface (where it attaches to the osteoblasts in cancellous bone) and does *not* depend on bone mineral density, it acts as a pharmacokinetic agent reflecting bone turnover and blood perfusion [4–7]. As ^18^F^−^ is rapidly taken up by bone, particularly healing bone, there is a high bone-to-background contrast [4], making it an excellent bone-imaging agent. In previous studies, values in the range of 200–600 HU were considered to represent cancellous/trabecular or healing bone while cortical (strong) bone is > 600 HU [8, 9]. The importance of ^18^F^−^ is that it shows bone formation or a lack thereof earlier than CT, indicating healing or no healing, thus enabling a determination of the state of the bone healing progress. If identification of patients with a high risk of delayed or nonunion could be done early, even preoperatively, it might be possible to avoid late revisions which lead to prolonged treatments and unnecessary late amputations.

Based upon our observations of the 41 patients in our cohort, in this report we propose to use histograms and cumulative distribution function (CDF) graphs derived from both the CT and PET data to evaluate bone healing and thus aid in the decision to revise a patient. We illustrate this with data selected from three of the six patients in our cohort who were successfully revised and went on to complete healing. This is contrasted with the data from a patient not needing revision. The proposed method extends the earlier analyses using spatiotemporal movies derived from the dynamic scans, static scans, and the PET uptake data [10–12]. The graphical methods introduced here and additional data, some of which are described in ESM 1 for each of our patients, could be used by the orthopedic surgeon to determine if the treatment should continue uninterrupted or if a course of intervention should be followed.

## Materials and methods

### Patients

Forty-one patients, 29 males and 12 females (mean age 44, range 17–78 years), who were treated with a TSF between October 2012 and October 2018, agreed to participate. Informed consent was obtained from all individual participants included in this study. The Regional Ethics Committee (Dnr 2012/1049-31/1) approved this study. There were no selection criteria, other than that the patient was able to come to the hospital for two PET/CT examinations and was willing to do so. Each patient had a complex tibia fracture and/or osteotomy. One patient had both tibiae treated simultaneously for Genu Varum; another had each tibia treated serially due to severe deformity from a previous accident making 43 tibiae treated. Ten patients had a second tibia break (nine osteotomies). Thirty-seven patients (38 tibiae) were examined at a mean of 56 days (range 40–148 days) after surgery and again at 105 days (range 81–188 days). Patient 2 was examined only once to determine the amount and spatial distribution of bone formation shortly before TSF removal, Patient 21 died between the first and second scans, and Patients 32 and 34 were examined only once as they were unable to come for their second scan. Of the six patients who were revised, five were examined a third time and two of these were examined a fourth time. A brief description of each patient and their condition is given in Table 1. A more extensive description of each patient is given in ESM 1.

**Table 1:** Patient description

Patient	Age	Sex	Days First PET/CT	Days Second PET/CT	Reason	Resolution	Days TSF applied
P1	64	M	274	N/A	Refracture in segmental tibia left	TSF extraction—not healed	328
P1	64		43	146	New TSF as fractures not healing	TSF extraction—healed	168
P1	64		374	400	New fracture between former two	Former two fractures remodeling	N/A
P2	36	M	135	N/A	Pseudarthrosis right lower tibia	TSF extraction healed	211
P3	52	M	40	84	Fracture healing in left tibia		167
P4	44	M	50	122	Pseudarthrosis right lower tibia—infection		161
P5	35	M	43	85	Genu Varum—pseudoachondroplasia		182
P6	17	F	52	94	Reduction malformation right tibia		345
P7	31	M	48	129	Osteomyelitis right lower tibia fracture	Leg amputated—continued infection	226
P8	28	M	60	184	Pseudarthrosis left lower tibia—infection	Patient did not heal—new operation	N/A
P8	28	M	288	363	Reoperated no new TSF was applied	TSF extraction healed—dancing	417
P9	45	F	50	91	Nonunion/pseudarthrosis distal tibia/pilon fracture right distal tibia	CT—nonunion-plane film X-ray *not* seen. Low 50 day uptake should have prompted revision	N/A
P9	45	F	224	294	Reoperated no new TSF was applied	TSF extraction healed	355
P10	33	M	42	90	Fracture varus deformity + lengthening		106
P11	68	F	43	87	Autologous bone grafting arthrodesis infection		156
P12	35	M	49	104	Severe bow deformities of tibiae—right tibia		151
	36	M	43	84	Severe bow deformities of tibiae—left tibia		184
P13	30	M	44	89	Varus deformity and lengthening		100
P14	21	F	48	94	Genu valgum—valgus deformity		115
P15	52	M	53	95	Pseudarthrosis—osteotomy infection	Patient not remodeling as expected	N/A
P15	52	M	148	N/A	Ongoing TSF with ultrasound of bone	TSF extraction—in cast	518
P16	40	M	145	184	Proximal tibia fracture—varus deformity—original scan delayed	TSF extraction healed—returned for a second scan 35 after removal	149
P17	70	M	48	82	Comminuted distal tibia fracture	TSF extraction healed	147
P18	29	M	44	83			199
P19	64	M	40	81	Proximal osteotomy; distal pseudarthrosis—infection		386
P20	58	M	42	83	Infected lower tibia arthrodesis—infection		173
P21	45	M	40	N/A	Fracture—infection	Died	57
P22	78	F	39	70	Distal tibia and fibula fracture accompanied by diabetes 85 mellitus	TSF extraction healed	128
P23	23	F	53	90	Pseudarthrosis—infection		109
P24	55	M	41	83	Arthrodesis—infection		153
P25	69	M	46	106	Pseudarthrosis—infection		252
P26	19	M	71	133	Posttrauma rotation deformity		149
P27	22	F	47	89	Tibia length discrepancy deformity		267
P28	23	F	47	69	Pseudarthrosis, ankle		112
P29	59	M	40	82	Open fracture distal tibia		174
P30	22	M	40	68	Pseudarthrosis infection	Patient did not heal—new operation	N/A
P30	22	M	172	N/A	Reoperation, no new TSF was applied	TSF extraction healed	228
P31	34	M	62	89	Trauma, open fracture—infection	Patient did not heal—new operation	N/A
331	34	M	179	N/A	Reoperation, no new TSF was applied	TSF extraction healed	390
P32	53	F	117	N/A	Diabetic neuropathy; failure of osteosynthesis		139
P33	46	M	109	153	Diabetic neuropathy; dislocation of fracture		173
P34	29	M	65	N/A	Knee dislocation; arthritis; arthrodesis		85
P35	40	M	73	122	Trauma acute shortening—lengthening proximal		142
P36	51	M	62	90	Open pilon fracture		164
P37	45	M	54	103	Diabetes obesity closed fracture		176
P38	72	F	61	81	Trauma redislocation—infection		116
P39	73	F	86	127	Pseudarthrosis—infection		131
P40	27	F	56	119	Open fracture; large segmental defect; new operation without removing TSF; no new scans	Distal TSF removed–healed Proximal TSF removed–healed	371 402
P41	69	M	61	103	Open fracture—infection	TSF extraction healed	280

Days are calculated from the surgery to attach the frame

*PET* positron emission tomography, *TSF* Taylor Spatial Frame™, *M* male, *F* female, *N/A* not applicable

### PET/CT scan

In this study, the salt Na^18^F which quickly dissociates into the Na atom and ^18^F^−^ ion subsequently carried throughout the body by the blood stream was used. To facilitate anatomic localization, three clinical PET/CT scanners (Biograph™ 64 True-Point™ TrueV, Siemens Medical Solutions, Erlangen, Germany; Discovery 710 and Discovery MI DR both from General Electric Healthcare, Waukesha, WI, USA) were used for the first 24 patient examinations, the next 15, and the last two, respectively.

The patients were hydrated with 70 mL of water before being placed supine on the scanning couch with both tibiae in the view as described in [13]. An anterioposterior scout view (CT topogram) was performed [13] to localize the crural fracture. This was followed by a diagnostic CT scan which was also reconstructed to be used for PET attenuation correction. The patient was then positioned in the PET scanner at the location of the crural fracture which included some or all of the TSF in the axial field of view so that only one bed position (22 cm Siemens; 15 cm GE) was required. A dynamic PET acquisition performed in list mode was started simultaneously with the intravenous Na^18^F injection. For the first 24 examinations 2 MBq [14] and for the last 17 examinations 1 MBq per kg body weight of Na^18^F were used to reduce the effective dose to the patient. To determine the increase in the absorbed radiation dose to the patient from the PET scan, we undertook a study, reported in [15], which showed that the increase was on the order of 0.5 mGy to the organ (bladder) most likely to be affected. The study also showed **no** increase in the CT portion of the scan. In addition, this study investigated using a radionuclide (^90^Sr) which is prevalent in very small quantities in the human body due to nuclear testing and accidents.

To study the spatiotemporal influx of radioactive material into the healing bone, the dynamic scan was reconstructed as a time series of volumes. The reconstructed series comprised six volumes at 10-s intervals (encompassing the first 1 min post-injection), four at 30-s intervals, seven at 1-min intervals, five at 3-min intervals, and four at 5-min intervals, totaling 45 min chosen in accordance with published guidelines [14]. In a previous study [10], PET volumes were reconstructed and compared at 30, 45, and 60 min, resulting in the 60-min reconstruction being found superior.

Additionally, a 5-min static scan was performed after 60 min [10, 13, 14]. In cases where there was a second break which was not in the original field of view, a second 5-min scan was obtained (*n* = 5/10). When the patient did not move, one noncontrast low-dose CT scan was used for attenuation correction of all reconstructions [13]. However, when the patient had moved between the dynamic and static scans (*n* = 4) or for patients requiring two 5-min scans (*n* = 5), a second CT scan was obtained, using a procedure identical to the first. The acquisition and reconstruction parameters for all scans are summarized in Table 2.

**Table 2:** PET and CT reconstruction parameters

Details				Resolution			Voxel size (mm)		
Modality	Model	Reconstruction	Type	*X*	*Y*	*Z*	*X*	*Y*	*Z*
PET	Siemens Biograph™ 64 True-Point™ TrueV	OSEM2D Four iterations Eight subsets Gaussian Filter 5 mm		168	168	74	4.07	4.07	3.00
	General Electric Discovery 710 and Discovery MI DR	OSEM Three iterations 18 subsets Gaussian Filter 5.5 mm		192	192	47	3.65	3.65	3.27
CT	Siemens Biograph™ 64 True-Point™ TrueV	120/140 kVp, 50/60 mAs, 0.5/1.0 s per revolution, 1.0 pitch	Attenuation correction	512	512	74	1.37	1.37	3.00
			Diagnostic	512	512	277/737	0.98	0.98	0.80/0.30
	General Electric Discovery 710 and Discovery MI DR	140 kVp, 60 mAs, 1.0 s per revolution, 1.0 pitch	Attenuation correction	512	512	47	0.97	0.97	3.27
			Diagnostic	512	512	241	0.97	0.97	0.625

### Image analysis

For intra-patient comparison of CT and PET volumes acquired at different times, the CT and PET data were spatially registered to bring the ends of the broken bones into alignment. A 3D image processing software tool, described and validated elsewhere [10, 16], was used. CT volume data from the subsequent examination(s) were spatially aligned to the CT volume data from the first examination by manually selecting physiologically guided landmarks on each tibia close to the crural fracture on the first diagnostic CT volume and then locating the matching points on each subsequent volume. From these landmarks, a registration algorithm created a rigid body transformation which brought each subsequent CT volume into alignment with the first one in a single coordinate system. Using numerous evaluation tools (2D and 3D, visual and quantitative), the landmarks were tuned until acceptable (less than 1 mm of misalignment). The transformation was evaluated by taking the landmarks on the volume to be aligned, and transforming them using the same transformation used on the entire volume. If the alignment were perfect, the landmarks would exactly overlap. The alignment was considered satisfactory if the three-dimensional distance between each set of corresponding landmarks was less than 1 mm. This same transformation was then applied to bring the subsequent PET volume(s) into alignment with the first one. As the original CT–PET alignment from some (*n* = 9/100) examinations was not perfect, the final CT–PET volume alignment was refined and evaluated with manual adjustments provided by the software. After alignment, the first CT and PET volumes were superimposed, and a 50-mm-diameter spherical volume of interest (VOI) was centered on the crural fracture region as visible on the CT volume, using a spherical landmark tool. This VOI was then transferred to all the aligned CT and PET volumes for that patient. The PET and corresponding CT from the subsequently generated volumes were superimposed to confirm the correct placement of the VOI in both PET/CT volumes. On PET volumes, VOIs were also placed on the contralateral tibia to include what was presumed to be normal bone. In the one patient who had both tibiae treated simultaneously, the normal bone VOI was placed on a portion of each tibia as far as possible from the crural fracture and any pins/wires from the TSF.

For semiquantitative evaluation of each PET study, the maximum and mean standardized uptake values (SUV_max_ and SUV_mean_) were calculated for each VOI [17] as well as the SUV for each voxel. For each CT study, the electron density in Hounsfield units (HU) was calculated for each voxel in the VOI. The complete voxel-by-voxel CT and PET data were recorded in a comma-separated values file. To make the semiquantitative data comparable for all patients with multiple examinations, the SUV_max_ and SUV_mean_ differences per day (SUV_max_DPD and SUV_mean_DPD) between the first PET/CT scan and each subsequent one were calculated. Patients who healed more rapidly between the first and second scans than between the operation and the first scan produced a negative SUV_max_DPD and SUV_mean_DPD. We used this SUV difference data to determine if it could be related to the duration of the bone healing. The SUV_max_, SUV_mean_, SUV_max_DPD, SUV_mean_DPD, and SUV_max_ from the contralateral tibia for each patient are given in Table 3.

**Table 3:** Summary of findings for all patients

Patient	Days post-TSF surgery	Operated tibia SUV_max_	Operated tibia SUV_mean_	Slope SUV_max_	Slope SUV_mean_	Nonoperated tibia SUV_max_
P1-Up	270	35.16	7.70			1.82
P1-Lo		18.95	5.50			
P1-Lo	43	35.05	7.98			2.40
	148	40.01	10.01	0.047	0.019	2.10
P2	133	23.59	6.94			1.34
P3	39	60.77	18.94			2.21
	83	48.03	14.46	− 0.290	− 0.102	2.46
P4	49	49.80	18.91			2.88
	119	33.57	13.24	− 0.219	− 0.079	1.92
P5-R	42	21.86	4.98			2.12
	83	40.36	5.42	0.440	0.010	4.86
P5-L	42	25.91	4.91			2.98
	83	30.56	6.62	0.111	0.041	4.31
P6	52	40.62	9.29			3.60
	94	34.64	7.11	− 0.142	− 0.052	2.92
P7	48	25.46	8.85			2.19
	129	22.40	8.65	− 0.038	− 0.002	2.36
P8	61	27.27	6.42			1.73
	183	27.91	6.50	0.005	0.001	1.26
	288	31.94	8.75			1.95
	363	20.88	4.21	− 0.144	0.059	1.07
P9	50	17.46	7.26			1.43
	91	28.33	10.53	0.265	0.080	2.13
	197	24.25	9.71			2.91
	269	19.38	7.32	− 0.068	− 0.033	2.80
P10	42	68.55	18.80			1.86
	90	65.61	23.79	− 0.060	0.102	1.04
P11	43	49.13	11.54			1.92
	87	24.84	6.81	0.362	0.280	1.18
P12-R	48	43.50	10.68			2.11
	104	27.68	10.90	0.292	− 0.009	2.30
P12-L	43	26.83	6.94			3.85
	84	30.12	7.05	− 0.0155	− 0.036	3.22
P13-Lo	44	17.58	3.61			1.38
	89	33.04	6.40	0.362	− 0.064	1.89
P13-Up	44	20.51	5.36			1.40
	89	18.04	5.07	0.057	0.007	2.30
P14-Lo	48	47.18	9.11			1.64
	94	29.29	5.23	0.374	0.084	1.28
P14-Up	48	70.26	9.32			2.87
	94	16.12	4.44	1.090	0.100	1.79
P15-Lo	53	31.91	9.15			1.43
	95	35.54	11.38	− 0.059	− 0.078	2.20
	148	20.54	7.37	0.112	0.012	2.69
P15-Up	52	26.37	7.95			
	93	28.16	6.41	0.068	0.046	
	148	20.00	4.45	0.013	0.071	
P16	145	31.63	10.18			2.92
	184	26.66	8.67	0.070	0.039	2.85
P17	48	29.62	10.00			2.97
	82	27.17	7.08	0.017	0.109	1.90
P18	44	14.29	3.59			1.01
	83	12.70	3.97	− 0.076	− 0.027	1.09
P19-Lo	40	26.53	11.53			1.98
	81	19.62	6.63	0.172	0.119	1.49
P19-Up	40	19.90	5.25			
	81	13.15	3.99	0.161	0.030	
P20	42	39.04	14.04			1.36
	83	35.94	11.75	− 0.727	− 0.035	1.34
P21	40	46.80	21.74			1.59
P22	39	26.90	9.56			1.59
	70	63.28	14.61	− 1.143	− 0.192	1.19
P23	53	27.86	8.48			2.98
	90	20.75	6.64	0.194	0.047	2.60
P24	41	25.31	13.77			1.07
	83	16.75	8.47	0.197	0.124	2.16
P25	46	31.60	6.48			1.71
	106	35.35	5.86	− 0.046	0.010	1.82
P26	71	57.40	11.49			1.10
	133	46.12	9.35	0.234	0.034	1.12
P27	65	40.23	4.72			1.33
	127	37.47	4.39	0.141	0.005	1.45
P28	47	50.51	8.04			1.62
	89	29.72	7.55	0.495	0.012	2.27
P29	40	13.70	2.83			1.03
	82	16.59	4.17	− 0.040	− 0.019	1.09
P30-Lo	40	15.32	3.91			1.18
	82	10.97	3.79	0.043	0.019	1.53
	172	22.83	4.75	− 0.049	− 0.008	1.79
P30-Up	40	34.54	7.56			1.83
	82	38.11	6.23	− 0.128	0.048	1.69
	172	29.59	8.54	0.037	− 0.007	1.72
P31-Lo	62	5.18	1.47			2.29
	89	8.71	1.87	− 0.739	− 0.065	1.39
	179	24.30	5.68	− 0.163	− 0.036	1.69
P31-Up	62	40.87	4.11			1.82
	89	17.48	3.72	0.866	0.014	1.76
	179	18.81	3.74	0.189	0.003	2.51
P32	117	68.01	11.91			1.93
P33	109	60.51	24.39			2.35
	153	57.88	13.04	0.060	0.258	2.11
P34	65	65.54	15.54			3.13
P35-Lo	73	41.56	4.17			2.07
	122	10.97	3.05	0.624	0.023	2.11
P35-Up	73	12.77	2.61			
	122	8.32	2.26			
P36	62	30.64	5.84			3.34
	90	21.77	4.26	0.317	0.057	1.80
P37	54	54.23	16.36			3.07
	103	81.05	20.38	− 0.547	− 0.082	1.98
P38	61	27.06	8.80			1.31
	81	28.04	7.05	− 0.187	0.055	1.83
P39	86	39.47	8.16			1.49
	127	27.39	6.76	0.295	0.034	1.75
P40-Lo	56	9.11	1.96			1.19
	119	12.92	2.71	− 0.054	− 0.010	2.29
P40-Up	56	29.22	6.40			1.52
	119	23.18	4.04	− 0.096	− 0.037	2.29
P41	61	26.26	5.64			1.61
	103	47.97	11.03	− 0.517	− 0.128	1.84

All SUV values for 5 minute scan after 60 min

Lo indicates distal tibia; Up indicates proximal tibia; L means left tibia and R means right tibia

All the voxels in each VOI were graphically displayed as histograms derived from both the CT and PET examinations to illustrate the difference between bone healing normally and that which was not, and to emphasize the value of the PET scan in assessing this. Additionally, we calculate a cumulative distribution function (CDF) for each VOI and show it graphically. For the 30 patients who had only two scans (no revisions) plus the two extra tibiae (Patients 5 and 12) making 32 points, a scatter plot of SUV_max_DPD versus time between the original operation and TSF removal was drawn. All graphical and numerical analysis was done using R version 3.2.3 [18].

## Results

All CT scans on the same patient were aligned to within 1 mm. Derived from the landmark data, this was considered to be acceptable. The PET scans were aligned using the same transformation.

As a first clinical example, we consider Patient 8, a 28-year-old man, who sustained a gunshot wound to the distal third of the tibia and fibula. After initial treatment with an intramedullary nail, he presented with an infected pseudarthrosis and a TSF was attached to his tibia. He had first and second PET/CT scans, both of which showed little progress toward healing. Since he was among our first group of patients, we did not immediately do a remediation. However, 246 days after attachment of the TSF, he was revised with a proximal osteotomy for tibia lengthening, bone grafts, and compression/stabilization of the nonunion. After the revision which did not include removal of the TSF, he had two more PET/CT scans, comparison of which showed progress toward healing. Patient 8 was healed and the TSF was removed after a total of 417 days.

**Fig. 1:**
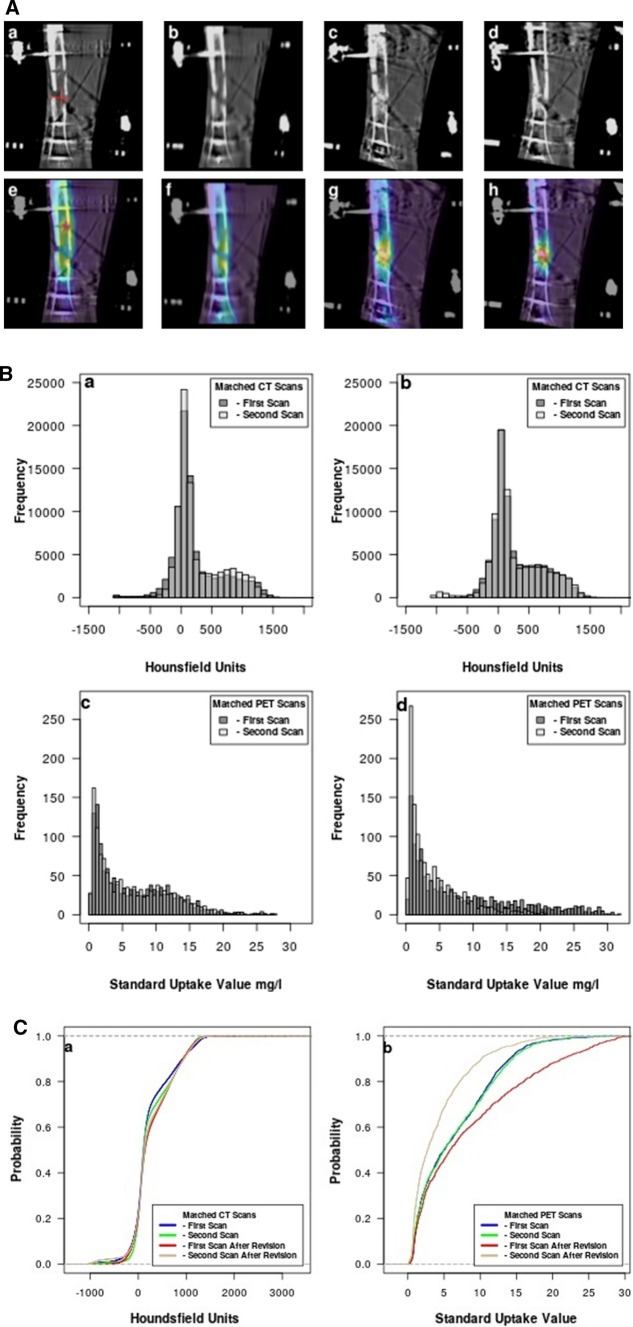
**A** The top row (a–d) shows a matched sagittal slice from the original CT scans of Patient 8 where (a, b) are before revision surgery and (c, d) are after revision surgery. A cross hair (a) marks the center of the VOI projected on that slice. The bottom row (e–h) shows the same sagittal CT slice projected onto the corresponding PET slice. The radionuclide uptake in (e, f) is seen to be unevenly distributed in the area of the crural fracture. It is more evenly distributed in (g, h) after the revision, showing progress toward healing. **B** CT histograms of VOI from the first and second scans of Patient 8 shown in (a) indicate that cancellous bone formation is very minimal. Histograms of the same region after the revision surgery are shown in (b). There seems to be more cancellous bone formation now, demonstrating progression toward healing. The histograms in (c) and (d) show the distribution of the radionuclide uptake before (c) and after (d) the revision. The increased radionuclide uptake is indicative of progress in healing. **C** The cumulative distribution function of the electron density (HU) for Patient 8 shown in (a) does not show much difference in between the CT scans, whereas the CDFs shown in (b) more clearly demonstrate a difference between the radionuclide uptake in the PET scans before and after revision

Figure 1A shows in the top row a matched static sagittal slice from the CT at the position of the crural fraction (a–d) from each of the four scans. In (a), a cross hair marks the VOI center projected on this slice. The second row (e–h) shows the CT slice from the top row superimposed on the matching PET slice. The first two columns (a, b, e, f) are before revision, and the last two columns (c, d, g, h) are after revision. The radionuclide uptake in (e, f) is seen to be unevenly distributed in the area of the crural fracture. After the revision, it is much more evenly distributed in (g, h), showing progress toward healing.

To quantitatively illustrate what is qualitatively seen in Fig. 1A, we show in Fig. 1B the CT and PET histograms of the VOI voxels. The first and second CT histograms as shown in (a) demonstrate very low electron density (HU) in the cancellous and cortical bone density regions. The third and fourth CT histograms after the revision (b) show some increased electron density in the range of cancellous bone, demonstrating progression toward healing. The first and second PET histograms (c) show little difference in uptake between the two scans, whereas after the revision, the third and fourth PET histograms (d) show a substantial increase in uptake indicative of healing progress. To further illustrate the difference in value between the CT and PET data, we show in Fig. 1C the CDF for the four CT scans in (a) and for the four PET scans in (b). There is very little difference in the CT electron density values, but a greater difference in the PET uptake values.

As a second clinical example, we consider Patient 36, a 51-year-old man, who sustained a complex fracture in a motorbike accident. In contrast to Patient 8, after the attachment of the TSF, he progressed rapidly toward healing with the apparatus removed after 164 days.

**Fig. 2:**
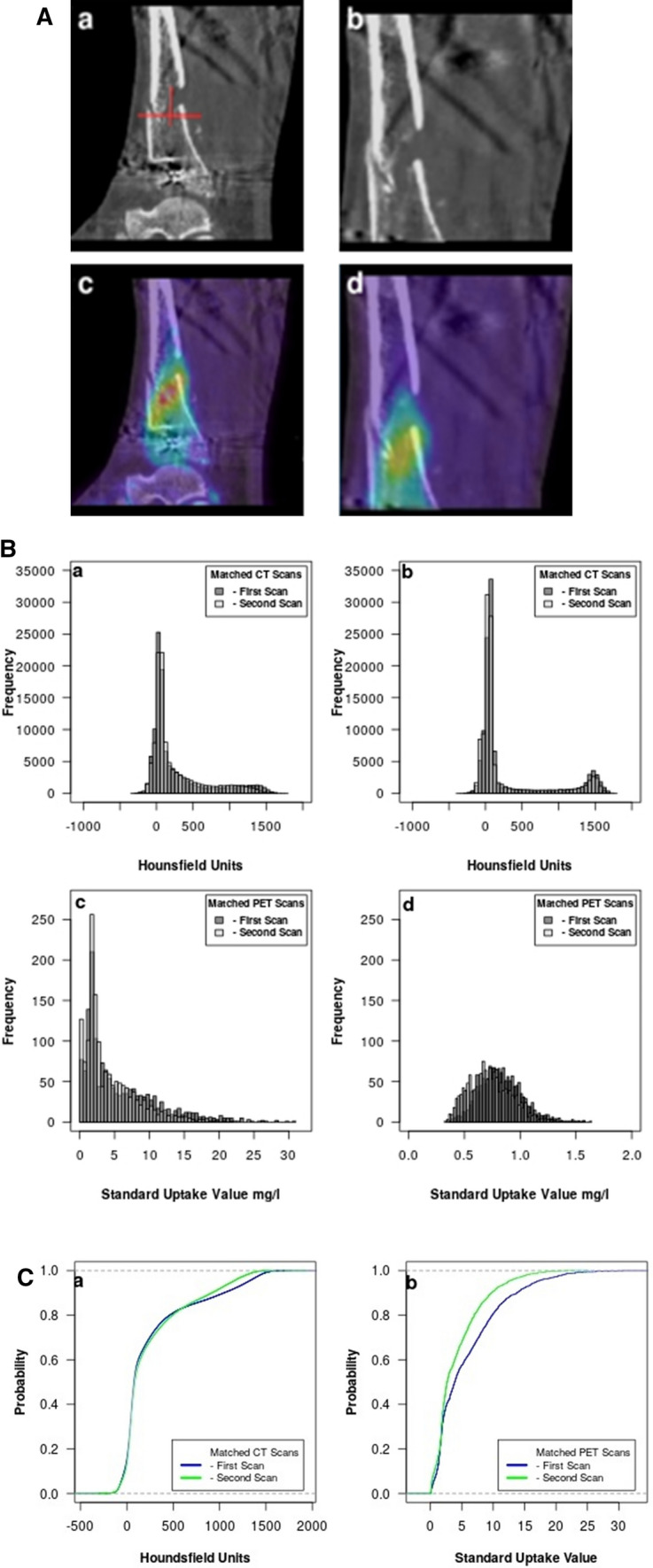
**A** This shows a sagittal slice at the level of the crural fracture through the first and second CT scans of Patient 36 in (a, b). A cross hair in (a) marks the center of the VOI projected on that slice. In (c, d), the CT slice is superimposed on the matching slice from the corresponding PET scan. The radionuclide uptake is seen to be evenly distributed throughout the crural fracture region. **B** Here, we compare the crural fracture of Patient 36 with the unaffected tibia. **B** (a) shows the CT histograms from the VOI in the region of the crural fracture. There is little difference between the two CT scans, but there is an indication of increased electron density in the cancellous bone region. However, in (b) which shows the electron density from the unaffected tibia, there are less electron density in the cancellous bone region and more in the cortical bone region. In (c), the radionuclide uptake in the region of the crural fracture indicates good healing progress. The radionuclide uptake in the unaffected tibia is shown in (d). Note the change in scale of the x-axis. The small amount of radionuclide uptake indicates normal bone turnover in contrast to healing bone (c). **C** The cumulative distribution function for the two CT scans in the region of the crural fracture for Patient 36 is shown in (a). In contrast, there is greater separation between the CDFs for the two PET scans shown in (b)

Figure 2A shows in (a, b) a matched static sagittal slice from the CT at the position of the crural fraction from each of the two scans. In (a), a cross hair marks the VOI center projected on this slice. The second row (c, d) shows the CT slice from the top row superimposed on the matching PET slice. It can be seen that the radionuclide is well distributed in the region of the crural fraction. Patient 36 had the same attributes in the healing process as Patient 8 did after the revision surgery.

Figure 2B compares the crural fracture with the unaffected tibia. In Fig. 2B (a), the first and second CT histograms demonstrate some electron density in the cancellous and cortical bone density regions and we can see that in the second scan there are more intermediate values—indicating an increased fraction of bone within the VOI. In comparison, the histograms from the CT scans of the unaffected tibia (b) show increased cortical bone and less cancellous bone. The first and second PET histograms, shown in Fig. 2B (c), indicate substantial radionuclide uptake in both the first and second scans. As expected for the unaffected tibia, the histograms for the PET scans (d) show very little difference between the two scans. Note the change in scale on the x-axis which highlights the difference in radionuclide uptake between healing bone (c) and normal bone (d). In Fig. 2C (a) which shows the CDF for the CT scans, it is easier to see there is little change in the fraction of voxels between cancellous and cortical bones. In Fig. 2C (b), there is a greater separation between the CDFs for the two PET scans.

As a third clinical example, we consider Patient 30, a 22-year-old man who had an infected nonunion. Bone resection at the infected site was performed together with a proximal osteotomy for bone transform, and a TSF was attached. After his second PET/CT scan, it was decided that docking surgery with bone graft after finishing the bone transport was indicated. This was done 138 days after attachment of the TSF. He then progressed rapidly toward healing with the apparatus removed after 228 days.

**Fig. 3:**
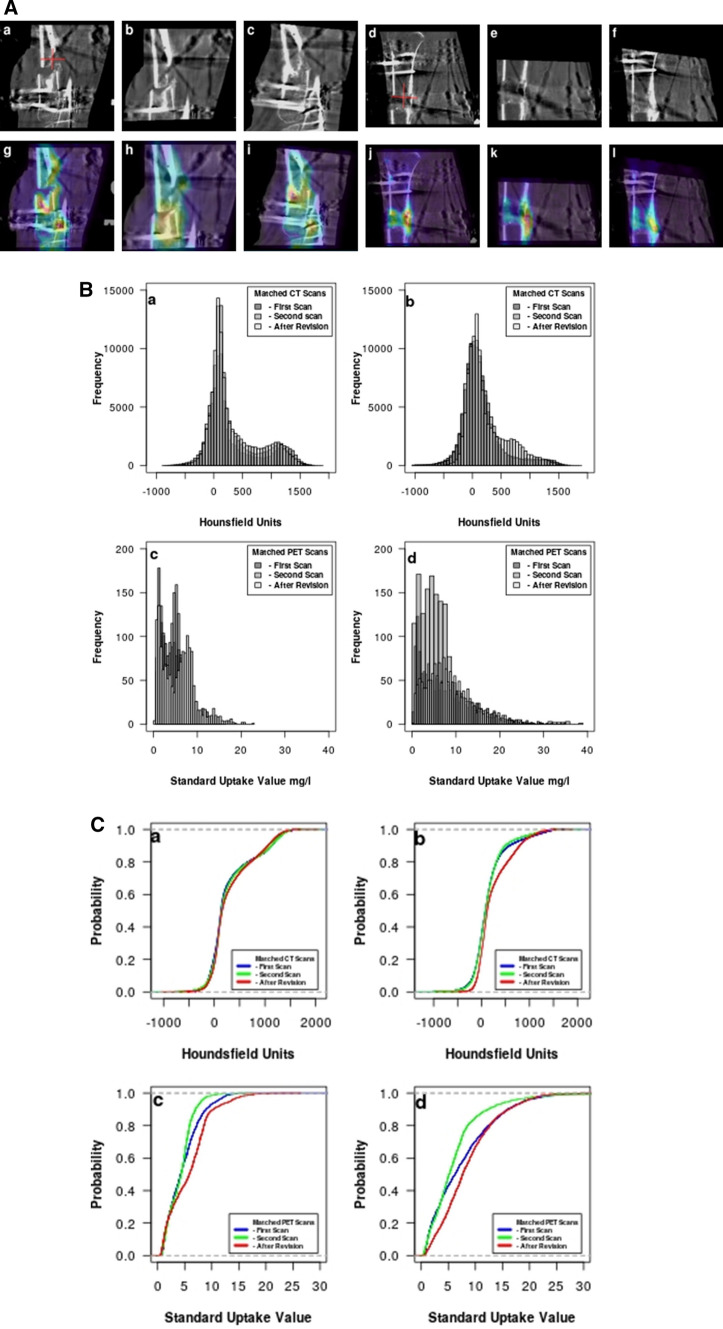
**A** In (a–f), matched sagittal slices from the original CT scans of Patient 30 are shown, where (a, b) are before revision surgery, (c) is after revision surgery, and (d–f) are the osteotomy. A cross hair in (a, d) marks the center of the VOI projected on that slice. In (g–m), the same sagittal CT slice is shown projected onto the corresponding PET slice. The radionuclide uptake in (g, h) is seen to be unevenly distributed in the area of the crural fracture and appears to be associated with the wire on the TSF. After the revision, it is more evenly distributed (i) showing progress toward healing. The radionuclide uptake in (j–l) is uniform in all the scans indicating good healing progress. **B** CT histograms from the VOI in the region of the crural fracture for Patient 30 are shown in (a) and from the osteotomy in (b). The superimposed histograms of the radionuclide uptake from the crural fraction (c) and the osteotomy (d) are also shown. There is greater uptake in (c) after the revision. **C** The cumulative distribution function for the CT scans for Patient 30 in the region of the crural fracture is shown in (a) and for the osteotomy in (b). The CDFs for the PET scans in the region of the crural fracture are shown in (c) and for the osteotomy in (d)

Figure 3A shows the sagittal view of the CT scan at the level of the crural fracture (a–c) and at the level of the osteotomy (d–f). The cross hair in (a) and (d) denotes the center of the VOI projected on that slice. The CT slice from (a–f) is superimposed on the PET scan at the level of the crural fracture (g–i) and at the level of the osteotomy (j–l). In Fig., (a, b, g, h) are before the revision at the level of the crural fracture. The radionuclide uptake in (g, h) is seen to be unevenly distributed in the region of the crural fracture and appears to be associated with the wire on the TSF. After the revision, radionuclide uptake is much more evenly distributed (i) showing progress toward healing. The radionuclide uptake in (j–l) is uniform in all the scans indicating good healing progress.

Figure 3B shows the superimposition of the histograms for the VOI for the three CT scans (a, b) and for the three PET scans (c, d). In (a) and (c), the histograms are for the crural fracture, whereas they are for the osteotomy in (b) and (d). After the revision, there is much greater uptake in Fig. 3B (c).

Again to more easily visualize the differences between the histograms, we compute the CDF for Patient 30 and show it in Fig. 3C. The CDF for the crural fracture CT scan is shown in (a) and for the osteotomy in (b). The CDF for the corresponding PET scans is shown in (c) and (d).

**Fig. 4:**
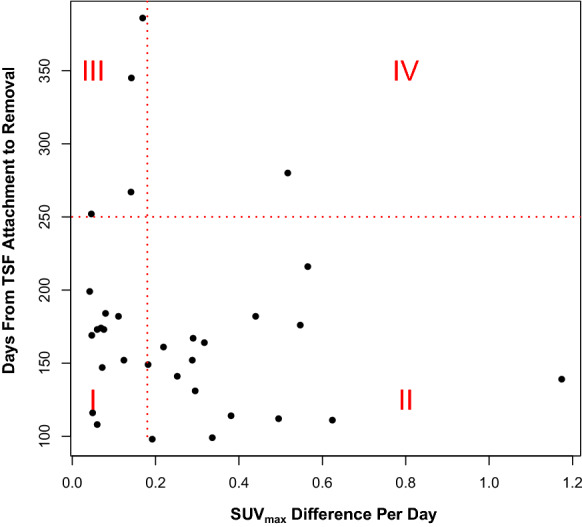
The scatter plot shows the standardized uptake value maximum difference per day (SUV_max_DPD) versus the days until removal of the TSF. It is divided into four regions which categorize the data shown in Table 3. Region I shows the patients with a SUV_max_DPD less than 0.18, but who achieved healing in less than 250 days. Region II represents patients with a SUV_max_DPD of 0.18 or greater and who achieved healing in less than 250 days. Region III represents patients whose SUV_max_DPD was less than 0.18 and who needed more than 250 days to achieve healing. There is one patient in Region IV where the SUV_max_DPD was greater than 0.18 and the patient took more than 250 days to heal

We present in Fig. 4 a scatter plot of the absolute value of the SUV_max_DPD versus time between the original operation and TSF removal. We have divided the graph into four regions, which seemed to reflect the relationship with the SUV_max_DPD and the number of days between TSF attachment and removal (Table 3). Based on the figure, there appeared to be a demarcation of the SUV_max_DPD value at 0.18 and of the removal time at 250 days. Of the 27 points on the graph which indicated rapid progression toward healing (less than 250 days before removal of the TSF), in 17 instances the SUV_max_DPD was equal to 0.18 or greater, but in ten instances it was less than 0.18. It should be noted that Patients 5 and 12 (both 35 year old men) have two tibiae represented. For the remaining five points, the bone healed in more than 250 days. One patient (Patient 41, a 69-year-old man) had a high SUV_max_DPD, but still took more than 250 days to heal possibly because he required two TSF pin adjustments.

## Discussion

Earlier we evaluated TSF treatment progression using CT [19]. Additionally, for many years, we investigated the use of CT together with 3D volume rendering techniques (VRT) to total hip arthroplasty (THA) [16, 20, 21]. The use of Na^18^F in bone scanning and the ready availability of cyclotron-produced Na^18^F lead to our earlier investigation of bone remodeling relative to THA using sodium ^18^flouride (Na^18^F) positron emission tomography (PET) [22]. The effect of metal artifacts in the CT examination on the PET attenuation correction was assessed, and a suitable reconstruction algorithm was determined via phantom studies [13]. Based upon these results, a suitable imaging protocol was designed and used in this study of whether Na^18^F PET/CT can help evaluate TSF treatment progression in a number of complex tibia cases. Hsu et al. [23] as well as Mathavan et al. [24] found that PET/CT was valuable in the evaluation of fracture healing in a rat model. Additionally, Mathavan also suggested that this method can separate bone formation from resorption and thus could be of interest across a wide array of orthopedic applications including as a predictive diagnostic tool to identify if fractures will heal successfully or result in delayed healing or nonunion. There is a review article which reviews techniques in limb lengthening and deformity [25]. As far as we can determine, our group is the only one using PET/CT bone scanning to evaluate patient treatment with a TSF. However, a recent review article gives a good overview of both diagnosis and treatment evaluation using PET/CT bone scanning of patients with osteoporosis [26].

In a very careful analysis, Du et al. [8] have shown that bone mineral density (BMD) is the determining factor to distinguish between cortical and cancellous (trabecular) bones and that the HU from CT scans is positively correlated with BMD. Other studies [27] have indicated that HU in differing area of cortical thickness may drop considerably in thin cortex areas because of the resolution limits and partial volume effect. Thus, the specific ranges of HU values associated with cortical and cancellous bones may vary with localized anatomy in different areas—making it difficult to assess bone healing. Schreiber et al. [9] have shown that there were significant correlations between HU and bone mineral density, age, and T-scores and also between HU and compressive strength. An earlier study showed a relation of mechanical properties in human bone to CT numbers and electron density [28].

In clinical example 1 in Fig. 1A (a, b), Patient 8 did not show much callus formation in the CT scans; hence, it would be difficult for the orthopedic surgeon to decide if the healing was going well. In contrast, when looking at Fig. 1A (e, f), one can determine that the radionuclide uptake is rather sparse and also unevenly distributed within the crural fracture. In Fig. 1A (c, d, g, h), we can see that after the revision surgery, healing seems to be taking place as there is some callus formation in (c, d) and the radionuclide uptake is evenly distributed in the crural fraction (g, h) showing progress toward healing. Although the CT histograms in Fig. 1B (a, b) show a slight increase in cancellous bone activity, in Fig. 1B (c, d) there is a great difference in radionuclide uptake seen in Fig. 1A (e–h) before and after revision surgery. The uptake decreases between the first and second scans after revision, as the rate of bone healing is decreasing with time. We have seen patients where the bone healing is very active in the first scan and drops off in the second scan and the reverse. Nevertheless, the difference in uptake between the first and second scans, particularly if the radionuclide uptake is uniform in the crural fraction region, is generally a good sign of bone healing. Although this patient might have healed eventually without the revision, we believe that he healed more rapidly after revision than he might have otherwise.

In clinical example 2 while Fig. 2A (a, b) shows vague signs of a callous forming, it can be seen in Fig. 2A (c, d) that the radionuclide uptake is well distributed in the region of the crural fraction. Patient 36 had the same attributes in the healing process, as seen in the PET scan, as Patient 8 did after the revision surgery. The histograms in Fig. 2B emphasize the difference between healing (a, c) and normal bone (b, d).

In clinical example 3, we show the fracture region for Patient 30 in Fig. 3A before (a, b, g, h) and after (c, i) revision as contrasted with the osteotomy. The callous formation in Fig. 3A (d–f) indicates that healing is occurring and the radionuclide uptake in (j–l) which is uniform in all the scans indicates good healing progress.

Although Fig. 4 shows a scatter plot of the standardized uptake value maximum difference per day (SUV_max_DPD) versus the days until removal of the TSF, we have not yet done an analysis to relate the healing time to the length of the gap to be filled—as has been done in earlier work by others who have calculated average healing index [2, 29, 30]. While this could be directly done for a osteotomy, it is not clear how this could be applied to fractures although some work has been done in [29]. This remains as future work.

**Fig. 5:**
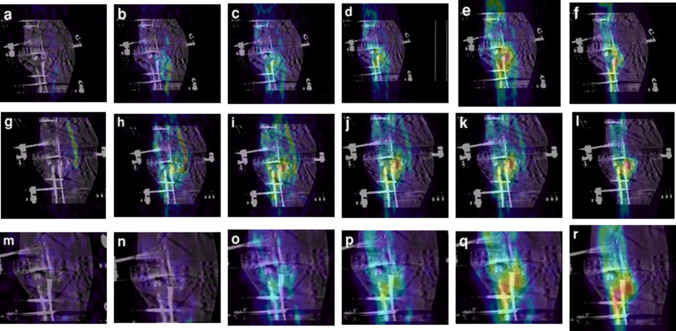
A sagittal slice from the CT scan at the level of the crural fraction for Patient 15 is shown superimposed on the corresponding PET slice. The columns represent the radionuclide uptake at the times 30 s, 40 s, 60 s, 120 s, 180 s, and 45 m after the initial injection. The first scan is shown in (a–f). After the first revision, the second scan is seen in (g–l) and the third scan after ultrasound stimulation is shown in (m–r). The radionuclide uptake is more uniform across the crural fracture in (m–r)

In addition to the examples provided in this report, we demonstrate the usefulness of the dynamic scan to show the spatiotemporal distribution of the radionuclide uptake in the following manner. We choose to illustrate this with Patient 15 who is a 52-year-old man with an infected pseudarthrosis. He was osteotomized proximally together with bone resection at the fracture site to correct a leg length discrepancy and varus deformity. His first PET/CT scan after 53 days, in Fig. 5 (a–f), shows that the radionuclide uptake in the crural fracture was minimal and not well distributed. Therefore, he was revised 79 days after the initial operation with a docking site refreshment and bone graft without removal of the TSF. After the patient’s second PET/CT scan at 95 days (g–l), as he still was not yet doing well, he was treated with ultrasound stimulation. Although this technique does not work in all patients, in this particular patient it seemed to be helpful as evidenced by his third PET/CT after 151 days (m–r) in which there are a more uniform distribution and greater uptake of radionuclide in the crural fracture. The patient was discouraged and spoke of amputation, but the orthopedic surgeon (CK–T) encouraged him to persist. The TSF was removed after 581 days from the initial operation, and after wearing a cast, the patient made a full recovery. ESMs 2–4 present the three movies associated with this patient.

In addition to the patients discussed here, the ^18^F-PET/CT scan was helpful in determining if the TSF could be removed. For example, in Patients 1 and 2, as discussed in ESM 1 where there is expanded information on all the patients, the PET/CT scan was confirmatory that the TSF could be safely removed for Patient 2 and negative for Patient 1. In Patient 6 who was osteotomized for leg lengthening, the fibular showed much higher activity on the first scan than the tibia and indeed it was necessary to re-osteotomize the fibular to obtain correct bone lengthening for the leg.

The limitations of this study were the small number of patients, which was exaggerated because the cohort was quite heterogeneous. Although there were 21 osteotomies, some were done for lengthening or deformity correction only and some were done for both. Ten patients had or developed pin infections, but this was not a major factor in the patients’ healing progress [12]. Our study was also limited by the fact that for most patients only two PET scans were performed. If additional PET/CT studies at 18 weeks and at 6 months could be done, these might provide more information, especially regarding predicting when the frame could be removed. While one dynamic scan acquired shortly after the operation, from which one movie could be produced, might give the surgeon an indication of good healing or not, this technique is quite new, and a larger cohort needs to be examined. To use the semiquantitative data in the form of the SUV, at least two PET/CT scans are necessary. However, use of the SUV as a definitive value is more problematic [17]. It is also true that a PET/CT is more costly than a CT scan alone, but if it can be shown that a PET/CT scan is valuable to the patient, especially in terms of early intervention if the healing does not appear to be progressing, or confirming that the healing has progressed to the point where the TSF can be removed without the possibility of the bone breaking again, then the benefits could outweigh the cost.

As future work, we would like to restudy patients with a clinical PET/CT bone scan close to the time of TSF removal to ensure that the bone is sufficiently healed (as was done for Patients 1 and 2). It also might be useful to perform an additional ^18^F^−^ bone scan after TSF removal to see if above-normal bone formation is still occurring. This was done for Patients 1 (210 and 236 days because of another fracture between the original two) and 12 (370 and 411 days in conjunction with the treatment of the left tibia). For these patients, an increase in radionuclide uptake in the VOI was seen indicating that the bone formation was occurring at a greater rate than expected for normal bone. In the last 17 patients in this study, we reduced the injected activity from 2 to 1 MBq per kg body weight without observing a reduction in the clinical information obtained. Thus, it should be possible to perform more scans during the course of the treatment. The value of this increase in the number of bone scans needs to be determined.

## Conclusions

We have attempted to show that in addition to CT, X-ray, and clinical examinations, a Na^18^F PET/CT bone scan may be helpful in determining progress in bone healing. Due to the heterogeneity of the VOI and the variance in the local bone remodeling, the use of simple descriptive statistics such as SUV_max_ and SUV_mean_ as well as the kinetic rate constants to analyze the static scans is insufficient. While the dynamic scans and the movies made from them help to capture the spatiotemporal radionuclide uptake, histograms and CDF graphs *based on all the voxels in the VOI* may be beneficial. Thus, ^18^F^−^ PET/CT bone scans might aid the orthopedic surgeon in assessing the patient’s progression to recovery. By identifying slow or insufficient progress at an early stage and understanding the uptake of ^18^F^−^ in specific regions of the bone, and then taking remedial action, it might be possible to shorten the recovery time and avoid unnecessary complications and amputations. This early identification of a need for intervention is the most valuable aspect of this technique. If we can take 3–6 months or more off the time in a circular frame, the cost of the PET/CT will soon be saved, and the patient can have an earlier return to his ordinary life. Using this method on every patient would probably be of value as we even in the uncomplicated cases sometimes encounter unexpected delayed healing. An indication that everything is looking good is also good information. However, as the method may increase the cost, at least in a short-term perspective, one might want to reserve the method to cases in which we can predict a problematic healing of either the fracture site or the osteotomy site. Apart from the clinical use, this method may also be used to evaluate different types of osteotomies, different devices for leg lengthening, and the benefit of different adjuvant treatments to support bone healing.

## Electronic supplementary material

Below is the link to the electronic supplementary material.

Supplementary material 1 (PDF 54 kb)

Supplementary material 2 (MP4 545 kb)

Supplementary material 3 (MP4 186 kb)

Supplementary material 4 (MP4 505 kb)
