# Supplementary material for: Can Na18F PET/CT bone scans help when deciding if early intervention is needed in patients being treated with a TSF attached to the tibia: insights from 41 patients
Source: Eur J Orthop Surg Traumatol. 2020 Sep 5;31(2):349–64. doi: 10.1007/s00590-020-02776-2 (PMC7875954; doi:10.1007/s00590-020-02776-2)
Supplement: Supplementary file 1 — Supplementary material 1 (PDF 54 kb) [file 590_2020_2776_MOESM1_ESM.pdf]

Can Na^{18}F PET/CT bone scans help when deciding if early intervention is needed in patients being treated with a TSF attached to the tibia: Insights from 41 patients

European Journal of Orthopaedic Surgery and Traumatology

Henrik Lundblad[1], Charlotte Karlsson-Thur[1], Gerald Q. Maguire Jr.[2], Marilyn E. Noz[3], Michael P. Zeleznik[4], Lars Weidenhielm[1]

Corresponding author: Lars Weidenhielm, Department of Molecular Can Medicine and Surgery, Karolinska Institutet, lars.weidenhielm@ki.se

Background information about patients

Patient 1 is a 64 year old man who had refractures in his open segmental tibia fracture. He was treated with fixation of both fractures in a two level TSF without revision. The first $^{18}F^{-}$ study was performed to aid the decision to extract the frame. The PET/CT indicated an ongoing high bone turnover indicating ongoing healing of the bone, confirmed by the morphological distribution of radionuclide uptake. The TSF was removed after 328 days at the patient's request, a cast was applied, but subsequently the patient had a refracture in the intermediate fragment and a varus dislocation that required further treatment with a TSF. The refracture was "activated" by drilling and a proximal osteotomy was done for gradual correction of the varus deformity and a slight lengthening. This second TSF was removed after 169 days. The patient then again developed a fracture in the intermediate segment that this time was treated with an intramedullary nail. For clinical reasons, he had two more PET/CT examinations at 374 and 400 days from attachment of the second TSF allowing the study of the ongoing tibia remodeling after removal of the TSF. He is doing well.

Patient 2 is a 26 year old man who was referred to us with a nonunion of a tibia fracture. The plate and a sequester of 5x2 cm was removed. He was then bone grafted and fixed in a TSF. He was painless in the frame but healing was very slow. Twelve months after the first operation a conventional x-ray showed healing of the fracture. At frame removal it was however evident that the fracture was unstable. The fracture site was therefore opened and revised and the frame was not removed. He went on to frame removal at 211 days after the fracture site was revised and has been stable after that.

Patient 3 is a 52 year old man who had a mal union of an open tibia fracture. This was originally treated with a nail and healed in varus and 4 cm of shortening after two reoperations and bone grafting. He was corrected and lengthened in a TSF with a distraction rate of 1 mm a day on the concave side. He showed poor bone regeneration and was therefore reoperated twice with stabilization of the frame and bone grafting. The patient was included in the study before the last revision. Due to previous failures this patient was also treated with at Physio Stim which is a device mimicking the electro-magnetic field of a healing fracture. The fracture healed and the frame was removed 167 days after the index operation.

Patient 4 is a 44 year old man who was referred to us because of an infected nonunion. A thorough revision and subsequent bone grafting and application of a TSF were performed. The patient refused extraction of the fibular plate and acute shortening which probably prolonged the course of treatment. His infection was successfully eradicated but the healing was very slow. He was included in the study before his last revision with bone grafting. The fracture went on to healing with frame removal 161 days after the index operation.

Patient 5 is a 35 year old man who had severely deformed and short limbs due to pseudo achondroplasia. He was included in the study when we performed bilateral proximal tibia osteotomies and application of TSF for lengthening and correction of severe varus and rotation. The correction was finished earlier on the right leg due to a residual deformity on the left side. Both frames were removed at 182 days.

Patient 6 is a 17 year old woman who had a reduction malformation of the right leg. She was operated with a TSF and a percutaneous osteotomy with a gigli saw of the tibia and an oblique osteotomy of the fibula with an oscillating saw. One week after the operation lengthening was started with a rate of 1 mm a day. Due to ankle contracture 5 weeks postoperatively she made a pause during 5 days in her lengthening. Two months postop we noticed that her fibula had healed prematurely and she was therefore reosteotomized. The distribution of the areas of maximum uptake was different in the tibia regenerate compared to the fibula. In the tibia we could observe three areas of high uptake whereas in the fibula there was only one area of maximum uptake. She went on to successful healing and the frame was removed 345 days after the initial operation.

Patient 7 is a 31 year old man who suffered a Gustilo3B open fracture to the distal tibia and was fixed first with a plate and screws. After three free flaps it was evident that he had a deep infection and was therefore operated with resection of dead bone, extraction of hardware and fixation with a TSF. A preoperative PET/CT might have indicated that more bone should have been resected. The low uptake at 6 weeks should have prompted an early revision and bone grafting. Recurrent infections lead this patient to decide in favor of an amputation.

Patient 8 is a 28 year old man who sustained a gunshot wound to the distal third of his left tibia and fibula. The fracture was fixed with an intramedullary nail and the patient presented five months later to the reconstruction section with an infected pseudarthrosis and a foreign body remaining in the soft tissue. He was revised with intramedullary reaming, extraction of the foreign body, application of Gentamycin, and fixation with a TSF. He was followed with plain film X-rays and was fully weight bearing and painless. However, a CT scan showed a hypertrophic nonunion. After 246 days from the original operation, he was revised with an osteotomy for lengthening of the tibia proximally, bone grafts, and compression/stabilization of the nonunion, without removal of the original TSF. He had the TSF removed at 417 days and commenced dancing lessons.

Patient 9 is a 45 year old woman who had a nonunion of the distal tibia after a pilon fracture that was fixed with plate and screws. She underwent hardware removal, revitalization (curettage) of the fracture site and fixation with a TSF. Standard x-ray was not able to identify any healing disturbances. However a CT scan clearly showed a nonunion. The low uptake at 6 weeks maybe should have been a signal that an early revision with autologous bone graft would have been beneficial to the patient. After 154 days from the original operation, she was revised with bone grafting, stabilization, and fibular osteotomy without removal of the original TSF. She had the TSF removed at 329 days.

Patient 10 is a 33 year old man who was otherwise healthy. He sustained a comminuted proximal tibial fracture after a gunshot wound in July 2009. He was initially treated with a temporary external fixator and a fasciotomy was performed. The wound was treated with vacuum assisted closure and the fracture was fixed with a bridge plate and thereafter a secondary suture of the wound. Due to delayed union the plate broke and was replaced in combination with an autologous bonegraft. In October 2012 he presented with increasing medial pain, 19 degree varus deformity and a leg length discrepancy of 15 mm. In August 2013 he underwent a high tibial osteotomy that was fixed in a circular frame, TSF, for gradual correction of the deformity. The patient was very distressed by the frame but went on to uneventful healing and the frame was removed after 108 days.

Patient 11 is a 68 year old woman with rheumatoid arthritis. After numerous operations of her left foot she was operated with an ankle endoprosthesis in her left ankle on September 02, 2013. Due to postoperative infection the prosthesis had to be removed and the foot and ankle were held in a temporary external fixator (Hoffman II). The infection and wound were treated with antibiotics and a VAC respectively. On October 12, 2013 she underwent autologous bonegrafting and a fixation in a circular frame, TSF. The frame was removed after 216 days. She is now fully weight bearing with minimal pain and the anterior wound has almost healed.

Patient 12 is a 35 year old man with hypophosphatemic rachitis. Due to a hydronephrosis he has a kidney failure and is under hemodialysis three times a week. He presented with severe bowdeformities of both tibiae and femora. At the time he was smoking 10 cigarettes a day. He was previously operated with a femoral osteotomy of his left femur which was fixed with an intramedullary nail with a residual varus deformity. We operated his right femur and tibia on December 12, 2013. The femur was fixed with an intramedullary nail whereas the tibia was fixed in a TSF for gradual correction. He went on to uneventful healing and after 152 days the frame was removed. His left leg tibia was subsequently fixed in a TSF on November 4, 2014 for gradual correction and he went on to uneventful healing after 184 days with both legs even in length.

Patient 13 is a 31 year old otherwise healthy man that sustained an open distal tibia fracture in a motor vehicle accident at the age of seven. The fracture healed with a severe s-shaped 20 degree varus deformity and a 70 mm shortening. The skin was adherent to the distal medial part of the tibia and ankle joint. On February 6, 2014 he was operated with a corrective osteotomy at the fracture site and a proximal osteotomy for lengthening. The distal osteotomy could not correct the deformity completely due to the skin condition. The lengthening is now completed and the frame was removed on May 16, 2014 after 99 days.

Patient 14 is a 21 year old woman from Gambia with multiple cartilaginous exostoses and genu valgum of 30 degrees and a valgus deformity also of the distal tibia of 20 degrees. On February 3, 2014 she underwent an acute correction of the left distal tibia, a resection of exostoses of her left distal femur and proximal tibia and fibula. During the same surgery we released the peroneal nerve and performed a proximal tibia osteotomy for

gradual correction of the valgus deformity. Both osteotomies were fixed in a TSF and have now healed uneventfully. The frame was removed May 28, 2014 after 114 days.

Patient 15 is a 52 year old man who presented with a pseudarthrosis which was fixed with a nail on January 14, 2014. On February 6, 2014, the nail was removed due to infection and he was fixed in an external Hoffman frame with cement and antibiotics. On May 12, 2014 the Hoffman frame was replaced by a TSF with removal of the cement and an osteotomy in the proximal tibia. On July 30, 2014 he underwent docking site refreshment and bone graphing without removal of the TSF. His progress was slow, therefore it was decided after his second PET/CT scan to use ultrasound stimulation to encourage bone growth. He had a third PET/CT after another six weeks. He went on to heal eventually with the TSF removed on October 12, 2015 after 518 days.

Patient 16 is a 40 year old otherwise healthy man that sustained an open proximal tibia fracture in April 2013. He was treated with an acute temporary external fixator, Hoffman II, and then fixed by percutaneous plating on April 17, 2013. He was randomized to receive hyperbaric oxygen treatment in a randomized controlled study. October 29, 2013 he still suffered from pain and radiologically there was a nonunion and a varus and procurvatum deformity of 10 degrees each. On March 13, 2014 he was operated with a proximal tibial osteotomy that was fixed with a circular frame, TSF, for gradual correction of the deformity. He went on to uneventful healing and the frame was removed August 12, 2014 after 152 days. He is now fully weight bearing with no pain. He had one PET scan before and the second after removal of TSF because of a scheduling delay.

Patient 17 is a 65 year old man with high blood pressure but otherwise healthy. On July 2014 he fell 2 meters from a ladder and sustained a comminuted intraarticular open distal tibia “pilon tibiale” fracture. He was temporarily stabilized in an external fixator, Hoffman II, which was exchanged for a circular frame, TSF, July 21, 2014. On a plain radiograph there is callus formation. He is now fully weight bearing with no pain or infection. He healed uneventfully and the frame was removed on December 15, 2014 after147 days.

Patient 18 is a 29 year old otherwise healthy construction worker who fell 8 meters on July 22, 2014. He sustained an unstable vertebral fracture of L2 with loss of sensory and motor function below his right knee, a wrist fracture and a comminuted intraarticular open distal tibia “pilon tibiale” fracture. The tibia fracture was immediately reduced in an external fixator, Hoffman II. The vertebral fracture was fixed with rods the same day after which he has gradually regained function in his right leg. The wrist fracture was reduced and held in a cast until July 27 when it was fixed with a plate and screws. On July 31 the external fixator was extracted and replaced by a circular frame, TSF. The patient gradually started to bear weight and was able to terminate taking any medication for pain. On a plain radiograph the healing of the fracture is progressing. The frame was removed on February 15, 2015 after 199 days.

Patient 19 is a 65 year old man that January 2013 sustained a wound from a chainsaw to the distal third of his left tibia. The wound was irrigated and revised in the operating theatre but shortly after this the tibia fracture was completed and subsequently fixed with a plate and screws. He soon developed a deep infection and mal union and in December 2013 he was operated again with removal of the plate that also by then was broken. After revision surgery there was a 2 cm bone defect that was filled with a cement spacer with Gentamycin. The tibia was now stabilized with a hybrid external fixator. February 2014 the cement spacer was removed and the bone defect was filled with autologous bone graft, according to Masqelet. The infection was however still present and the bone graft therefore was reabsorbed. The patient was referred to us and October 23, 2014 he was operated with a resection of all dead bone and a proximal osteotomy for a subsequent bone transport in a TSF. The bone transport was uneventful but the callus formation slow leading us to perform an autologous bone graft to the regenerate at the distraction osteotomy and a percutaneous drilling of the docking site. This was done in May 2015. He then went on to bony union and the infection was eradicated. The frame was removed November 13, 2015 after 386 days.

Patient 20 is a 59 year old man who suffered from a mono arthritis in his left ankle. For this reason he had two surgeries. One in 2008 and in March 2014 he underwent a subtalar arthrodesis. After this he had a fulminant infection that was drained in Italy where he spent his summer vacation. On August 14 he presented at our emergency ward at Karolinska University Hospital and two days later he was operated with a revision surgery and removal of all hardware. Four days later he was stabilized in an external fixator and vacuum assisted closure (VAC) was initiated. After changing the VAC a few times he was again operated on September 19, 2014 when the bone defect was revised and filled with a cement spacer with Gentamycin. November 5, 2014 the external fixator of Hoffman type was changed to a TSF. We removed the cement spacer and filled the defect with a

mixture of autologous bone graft and calcium sulphate mixed with Tobramycin and Vancomycin. After a minor operation because of a pin infection in the forefoot the frame was removed in April 27, 2015 after173 days. The infection has now subsided and he has a stiff painless ankle. He is due for an x-ray within 6 months to confirm complete consolidation.

Patient 21 was a 45 year old man with obesity, high blood pressure, high blood fats, posttraumatic stress syndrome and depression due to recent loss of his wife and sister. He was admitted to the ER/trauma room on June 10, 2014 after an assault where he sustained an open fracture of the distal tibia and fibula (Gustilo-Anderson II). He was immediately taken to the OR for debridement and fixation with an external fixation (Hoffman). The wound was sutured and after recovery of the skin and soft tissues the external fixator was removed and the fracture fixated with MIPO (minimally invasive plate osteosynthesis) on June 27, 2014. Postoperatively he was treated with p o antibiotics (Flucloxacillin and later Clindamycin) because of discharge from wound. After removal of antibiotics he was admitted again on September 1, 2014 for debridement surgery because of fever, pain in the leg, local severe swelling, redness and discharge: After reoperation he is again treated with p o antibiotics. Due to persistent discharge and later breakage of plate and screws, he underwent new surgery January 15, 2015 where all metal is removed, bacterial culture samples were removed and the infected pseudarthrosis was filled with calcium sulphate mixed with Gentamycin and the pseudarthrosis is stabilized with a TSF. Because of lack of appropriate stability a foot plate was added to the frame January 31, 2015. The patient was given one PET scan but then lost to follow up as he was found deceased in his home 57 days after the TSF was attached.

Patient 22 is a 78 year old lady with high blood pressure and insulin dependent diabetes mellitus since several decades. She was admitted to the ER January 21, 2015 after slipping on ice where she sustained an open fracture of the distal tibia and fibula (Gustilo-Anderson I). She was initially treated with external fixation (Hoffman). Due to of the nature of the fracture (open fracture), soft tissue conditions, and comorbidity (Diabetes Mellitus), the initial external fixator was changed to fixation with a TSF on January 29, 2015 to improve stability and enable weight bearing. Soft tissues healed uneventful. The fracture healed and the frame was removed June 17, 2015, after 139 days.

Patient 23 is a 24 year old medical student who suffered an open comminuted pilon fracture (distal tibia) and a non-displaced calcaneal fracture on April 16, 2014. The pilon fracture was operated elsewhere and was left with an unclosed wound anteriorly because of skin tension. As she returned to Sweden in May the wound was covered with a partial skin graft after treatment with vacuum assisted closure (VAC). The skin recovered but she developed a pseudarthrosis (nonunion) in the distal tibia and a posttraumatic osteoarthritis in the ankle joint. On February 9, 2015 she was therefore operated with removal of the plate and screws, autologous bonegrafting and Stimulan ($CaSO_4$) mixed with Vancomycin. The fracture was fixed in a TSF and the ankle was distracted (arthrodiatasis) in order to improve the pain from arthritis. The nonunion healed in the frame which was removed May 18, 2015 after 98 days. She still suffers from ankle pain due to osteoarthritis but the fracture is radiologically united.

Patient 24 is a 55 year old man who was otherwise healthy. In March 2012 he was mountain climbing in Turkey and fell to sustain an open comminuted fibular fracture. He was transported with a helicopter to a hospital where the wound was cleaned and the fracture was stabilized with an external fixator. On returning to Sweden the external fixator was removed and the fracture reduced and fixed with a plate and screws on April 2, 2012. Two months later he was due to remove a syndesmosis screw. Upon suspicion of delayed union the screw was left in place. One month later he presented with a fracture dislocation and a subluxed ankle joint. For some reason there was now also a pilon fracture that either earlier was non-displaced or was caused by intensive mobilization of this highly motivated patient. The fractures were again fixed with plates and screws. He was then revised twice because of infection and on May 6, 2013 he was operated with removal of plates and screws and an arthrodesis with distal fibula as an autologous bonegraft and fixation in a TFS. The patient was fully weight bearing and the frame was removed July 25, 2013. The patient was put in a cast for one month and was then back to normal activity. Unfortunately the arthrodesis gradually displaced into almost 30 degrees of valgus and because of this he was again operated March 15, 2015. We did a closing wedge osteotomy and mixed the wedge with Stimulan mixed with Vancomycin. The osteotomy was again fixed with a TSF which was removed August 18, 2015 after

156 days. The patient has a pain free arthrodesis and is very active and walks in the forest during hunting seasons.

Patient 25 is a 69 year old overweight man with hypertension and hyperlipidemia who sustained an open contaminated pilon fracture (Gustilo 3B) after a 3-meter fall from a ladder on July 5, 2016. He had a positive culture with mixed bacterias and a clinical infection. He was put in an external fixator and was put on long-term antibiotic treatment according to the culture. After several surgical revisions the wound was closed with a free gracilis flap 10 days after the trauma. Two days later he was put in a circular frame, TSF on July 28, 2016. Five months later he was taken to the operating room (OR) to refresh the fracture site by percutaneous drilling and injection of bone marrow aspirate from the iliac crest. After another 2.5 months he was again taken to OR to test the stability. It was decided that it was too early to remove the frame which instead was stabilized. Two months later on April 6, 2017, the frame was removed and the fracture as well as the infection healed.

Patient 26 is an otherwise healthy 19 year old man, who presented with a malunion of his right tibia after a fracture that was treated in a cast three years prior to corrective surgery. There was no infection, but the malunion of the tibia lead to an osteotomy and fixation with TSF on August 30, 2016 to correct a slight valgus and external rotation. After five months the frame was removed on January 26, 2017, and the patient was put in a cast for 4 weeks. He has had a complete recovery.

Patient 27 is a 22 year old otherwise healthy woman with a posteromedial angulation of her right tibia. She was treated at the children's orthopaedic department with physeodesis on the contralateral knee to correct the leg length discrepancy and staples to correct the deformity. She presented at our clinic at the age of 22. She was then complaining of pain and reduced dorsiflection of her right ankle. We found that she had a remaining LLD of 3 cm and a valgus and procurvatum deformity of 15 degrees each. She had a gradual correction in a circular frame, TSF, after a supramalleolar osteotomy on September 5, 2016. After lengthening she had a percutaneous achilles tendon lengthening due to contracture. Due to delayed union of the distraction osteotomy she was given Zoledronic acid at two occasions. After 9 months the fixator was removed on May 30, 2017, and the patient had regained good function and is walking without pain.

Patient 28 is a 23 year old woman who sustained multiple injuries in a motor vehicle accident, MVA 2014. She presented at our clinic with a posttraumatic osteoarthritis in the ankle joint. She underwent an arthrodesis with screw fixation but did not heal. Due to this pseudarthrosis she was operated on December 1, 2016 with a circular frame, TSF and an autologous bone graft. The frame was stabilized in OR after 2.5 months and after almost 5 months the arthrodesis was healed and the frame could be removed successfully on March 23, 2017.

Patient 29 is a 59 year old otherwise healthy male construction worker who was injured by an excavator. He sustained an open tibia pilon fracture that was treated acutely in a circular frame, TSF on December 8, 2016. There was no infection. After 5 months he was taken to OR in order to remove the footring and to stabilize the distal tibia ring. One month later the fracture was healed and the frame could be removed on May 31, 2017. The patient is walking without aids and apart from a reduced range of motion he has good function.

Patient 30 is a 22 year old man who sustained an open tibia fracture that was operated with plate and screws. He suffered a postoperative infection. He was referred to us from another hospital and taken to OR where the plate and screws were removed, the wound was revised, and he was put in a temporary external frame (Hoffman) and treated with long term antibiotics according to cultures. After four revisions and vacuum treatment he was operated with a bone transport (proximal corticotomy) in circular frame, TSF on January 10, 2017, implantation of bone substitute (Cerament with gentamycin) and a free gracilis flap. On March 28, 2017, after 3.5 months, the docking site was refreshed and grafted with autologous bone. After 7 months the fracture as well as the infection was healed, and the frame was removed on July 28, 2017. The patient is now walking and running without pain.

Patient 31 is a 34 year old man who sustained an open distal tibia fracture with bone loss after a firework blast injury. The injury was revised and treated in a vacuum dressing until definitive surgery 9 days (February 2, 2017) later with a free gracilis flap, proximal corticotomy for bone transport and application of a TSF. Four months later on June 1, 2017 the docking site was refreshed and grafted with autologous bone. Due to pin

infections the patient was taken to OR again one month later to exchange some of the pins that had been removed in clinic. Thirteen months after the injury the frame was removed on March 8, 2018.. The patient was a smoker and kept smoking although less frequently throughout the treatment. Apart from some stiffness of the achilles tendon the patient is now walking and running without pain.

(8,JS) Patient 32 is a 53 year old woman with diabetes mellitus, hypertension and hypothyreosis who sustained a distal tibia fracture after a fall. She was operated with plate and screws but unfortunately the fracture was redislocated and the plate broke. She was therefore reoperated with extraction of the plate and screws and put in a circular frame, TSF on April 13, 2017. Peroperative cultures did not show any infection and after 5 months the frame could be successfully removed on August 30, 2017. She had some pin site infections and was taken to OR to exchange pins but otherwise the treatment was without complications. The patient has complaints about cold feet but it can probably be explained by her diabetes.

Patient 33 is a 46 year old man with Diabetes type 1 since childhood. He has multiple complications following diabetes such as retinopathy, neuropathy, and kidney failure. He also has a right side drop foot. Preinjury, he was able to walk without aid. He was admitted after a fall with a right closed tibia/fibula fracture. Initial surgery was performed with open reduction and plate fixation. The patient was readmitted 3½ months following index surgery, with fracture dislocation into varus and bent plate. On April 22, 2017 the plate was extracted and TSF was placed following osteotomy for sequential correction. The patient was allowed weight bearing as tolerated and full range of motion of the knee- and ankle joint when soft tissues had healed. There was no infection. The patient did not undergo any further surgery before removal of frame, which was performed on Oct 12, 2017 after 6 months of treatment.

Patient 34 is an otherwise healthy 28-year-old man that suffered a femoral fracture in a motor vehicle accident several years ago. He had a posttraumatic subluxation and osteoarthritis of his left knee and a leg length discrepancy of almost 5 cm. An arthroplasty was considered impossible and he therefore underwent an arthrodesis with a circular frame, TSF on June 18 2017. There was no infection. The frame was removed after 3 months on August 12, 2017. The knee was then pain free but he was still complaining about his leg length discrepancy.

Patient 35 is a 40 year old man with no previous medical history, who was referred after a motorbike accident. He sustained a right open distal tibia/fibula fracture as well as a right open calcaneal fracture. The fractures were held with a temporary external fixator (Hoffman) and revised twice before fixation on June 6, 2017 with a TSF frame. Acute shortening of the distal tibia/fibular fracture with wound closure and proximal tibia osteotomy for bone lengthening was performed. The calcaneal fracture was also fixated with the TSF. The patient was not allowed weight bearing for the first eight weeks. Three month after the TSF operation, the patient was readmitted for drilling of the fracture site (tibia) as well as removal of the calcaneal fixation (foot ring). The remaining TSF was removed on November 9, 2017, after a total of 5 months.

Patient 36 is a 51 year old man who was admitted following a motorbike accident. He was a smoker before the injury. He sustained multiple injuries, among them was a left open tibia/fibula Pilon type fracture. The injury was temporarily fixated with an external fixator (Hoffman) and treated with repeated revisions (n=3). Definitive surgery with TSF was delayed due to thoracic injuries, pulmonary embolism, and pneumonia/sepsis. The TSF surgery for definitive fixation and wound closure was undertaken on September 12, 2017, 3 weeks following the injury. The patient did not weight bear fully until 3 months postoperatively due to his other injuries. The foot frame was removed after 3 months and the remaining frame on February 23, 2018 after a total of 6 months.

Patient 37 is 54 year old man with Diabetes type 2 and neuropathy, obesity (BMI 43), hypertension, and hyperlipidemia. He was referred following a closed left tibia/fibula fracture due to a fall. The skin around the fracture was severely bruised. A TSF was applied December 14, 2017 and included the ankle joint due to fissure down to the ankle joint. The patient was initially partially weight bearing (8 weeks), and after that weight bearing as tolerated. The ankle joint fixation was removed after 3 months. The patient was admitted through ER because of pintract infection after 4 months of frame treatment, when one pin as well as one wire is removed,

together with treatment of Cloxacillin (i v) followed by Flucloxacillin (p o). The infection healed uneventfully and the remaining frame was removed on June 9, 2018 after a total of 6 months.

Patient 38 is a 71 year old woman with substitution of thyroid hormone (Levaxin), who sustained a closed left pilon type ankle fracture with a large posteriomedial fragment due to a fall. She was initially treated with an open reduction and plate fixation. Due to fracture dislocation and varus deformity she was readmitted after four weeks. On December 21, 2017 the plate was extracted, osteotomy of the fracture site to enable gradual correction was done, and a TSF (including the ankle joint) was applied. The system was considered stable and the patient was allowed full weight bearing. The foot ring was removed after 2 months and the remaining frame on April 16, 2018 after a total of 4 months

Patient 39 is a 71 year old woman with rheumatoid arthritis, treated with Methotrexate and Orencia. She has a drop foot on the right side, due to spinal stenosis. She was admitted with a right open lower leg fracture after a fall while walking outdoors. She was treated with revision of wound and temporary external fixation (Hoffman), after which the fracture was treated with open reduction and plate fixation. After the surgery she was treated with oral antibiotics because of wound discharge, and after 3 months a surgical revision including microbiology cultures was performed showing Staphylococcus aureus. She was continuously treated with Flucloxacillin (p o). After one year the plate was removed. The fracture then showed only partial healing with a defect resulting in varus instability. Therefore the patient was treated with thorough surgical revision of wound and bone and instillation of injectable calcium sulfate/hydroxyapatite with Gentamycin (Cerament G) together with fixation with the TSF on December 3, 2017. Full weight bearing was allowed. Microbiology cultures still showed Staphylococcus aureus. The TSF treatment period was uneventful and frame was removed on April 13, 2018 after 4 months. Antibiotics (Flucloxacillin) were applied until the skin healed.

Patient 40 is a 27 year old woman, no previous medical history, who sustained a left open tibia/fibula fracture due to a motorbike accident. She was treated with wound revision and temporary external fixation (Hoffman). Due to severe soft tissue damage she was initially planned for a free muscular flap (latissimus dorsi) and nailing. Perioperatively, substantial bone loss was observed and fixation treatment was changed to bone resection at fracture sight, proximal osteotomy (tibia) and fixation TSF for bone transport on June 25, 2018. Bone loss was about 8.5 cm. Bone transport followed for about 3 months, when she was allowed partial weight bearing. After that, on October 23, 2018 docking surgery was performed with an autograft from the iliac crest (left). During the three months of bone transport, she was admitted once following a pin tract infection. The pin was removed and she was treated with antibiotics. After the docking surgery full weight bearing was permitted. Due to the long bone transport segment the distal part of the TSF was removed on 1 July 2019 and the proximal part on 1 August 2019 after a total of 18 months.

Patient 41 is a 69 year old man with treatment because of high blood pressure. He was referred following a motorbike accident when he sustained a right open tibia/fibular fracture. Temporary external fixation (Hoffman) as well as a series of surgical debridements had been performed at the referring hospital. Due to substantial loss of surrounding soft tissue, the patient was planned for a free muscular flap. Fracture fixation with TSF on June 27, 2018 was chosen because of the long time in external fixation before soft tissue coverage (4 weeks) and colonization with Enterobacteriaceae. This was treated with oral antibiotics; Bactrim forte. Perioperative cultures also showed Staphylococcus epidermidis, which was treated with Linezolid. The patient was allowed weight bearing as tolerated. During the treatment time the patient was admitted for surgery for pin adjustment on two occasions. He was diagnosed with deep vein thrombosis and treated with Low Molecular Weight Heparin (LMWH). The wound healed and the frame was removed on April 3, 2019 after a total of 9 months.
